# Survival Outcomes in Older Palliative Care Patients With Impaired Swallowing Function Receiving Only IV Fluid Support: A Retrospective Observational Study

**DOI:** 10.7759/cureus.107463

**Published:** 2026-04-21

**Authors:** Yuhei Nagaoka, Ryosuke Hamanaka, Kenji Umeki, Kosaku Komiya

**Affiliations:** 1 Department of Respiratory Medicine and Infectious Diseases, Oita University Faculty of Medicine, Yufu, JPN; 2 Department of Respiratory Medicine, Tenshindou Hetsugi Hospital, Oita, JPN

**Keywords:** end-of-life care, nil per os, percutaneous endoscopic gastrostomy, survival duration, swallowing dysfunction

## Abstract

Objective* *

This study aimed to describe survival after initiation of nil per os (NPO) in older adults who lost swallowing function and were managed with IV fluids alone, and to examine differences according to terminal clinical course.

Patients and methods

This retrospective study included patients hospitalized with pneumonia (April 2015-March 2025) who recovered from pneumonia but developed irreversible swallowing impairment, could not take food orally, were managed under an NPO policy, and died during the same hospitalization. Patients were classified into three groups according to the principal cause of death: senility, recurrent pneumonia, and other causes. Patients who died before recovery from the pneumonia that led to hospitalization were excluded. The primary outcome was days from NPO initiation to death, and secondary analyses compared clinical and laboratory characteristics among groups.

Results

A total of 255 patients were identified during the 10-year study period through initial screening, and 68 were included in the analysis after excluding 183 patients who died before recovery from the pneumonia that led to hospitalization and 4 patients who had received high-calorie parenteral nutrition during hospitalization. These patients were classified according to the cause of death into a senility group (n = 33), a recurrent pneumonia group (n = 24), and an other-cause group (n = 11). Median survival after NPO onset differed among groups (p < 0.01) and was significantly longer in the senility group (30.0 days, 19.0-47.0) than in the recurrent pneumonia group (14.5 days, 7.5-17.0). Baseline clinicodemographic characteristics did not differ among groups, except for sex; BMI and WBC counts tended to be lower in the senility group.

Conclusions

Among older adults receiving IV fluids alone after loss of swallowing function, survival after NPO initiation differed according to terminal clinical course, with the longest survival observed in the senility group.

## Introduction

Global physiological decline near the end of life is frequently accompanied by loss of swallowing function and aspiration pneumonia. For instance, reports from Japan indicate that more than 80% of patients with pneumonia aged ≥70 years are diagnosed with aspiration pneumonia [1]. A major contributing factor is impaired swallowing function, which is frequently associated with cerebrovascular disease, dementia, and being bedridden, and often reflects global physiological decline [2]. When recovery of swallowing function is unlikely, clinicians must decide whether to introduce enteral nutrition, such as via gastrostomy, or to provide parenteral nutrition in addition to pneumonia treatment [3]. The American Academy of Hospice and Palliative Medicine states that enteral feeding at the end of life is a medical intervention and that its initiation, continuation, or discontinuation should be determined according to treatment goals and the clinical context [4].

Percutaneous endoscopic gastrostomy (PEG), introduced in the latter half of the twentieth century [5], provides a safer and less invasive method for long-term tube feeding than open surgical gastrostomy. However, its benefits for frail older adults-particularly those with progressive dementia-have been questioned. Multiple studies have reported that enteral feeding does not clearly prevent aspiration pneumonia or prolong survival, and evidence for improvements in quality of life (QOL) with nasogastric tube (NGT) or PEG feeding remains limited, in part because of the risks of procedure-related complications [6, 7]. Therefore, clinicians, patients, and families face a difficult dilemma when choosing end-of-life nutritional care.

Feeding decisions must be aligned with patient values and goals of care, and there is an ongoing ethical and clinical debate surrounding tube feeding in this population [8, 9]. Therefore, patients and families are turning with increasing frequency to advance care planning (ACP), particularly in Western countries, and the higher prevalence of ACP has shifted the focus of decision-making from life prolongation to QOL and goal-concordant care. In Japan, enteral nutrition was traditionally initiated proactively when oral intake became difficult, and as a result, PEG use increased substantially in the decades following its introduction [8]. However, the debate surrounding PEG for terminally ill patients intensified, and in 2012, the Japan Geriatrics Society asserted that tube feeding should not be selected as a matter of routine; rather, decisions on tube feeding should be based on prognosis, patient preference, and the potential for improved QOL [10]. Following this 2012 position statement, the use of PEG gradually declined in Japan. By 2015, this downward trend was already evident, and PEG was no longer routinely performed, particularly in frail older adults. Instead, nutritional management shifted toward a more cautious and individualized approach, with increasing use of alternative strategies such as IV hydration. While wider ACP implementation has contributed to a gradual decline in new PEG placements, this shift raises the question of how nutritional care should be managed when neither NGT feeding nor gastrostomy is provided. Artificial nutrition is regarded as a medical intervention, and its withdrawal at the end of life may be ethically and legally accepted as part of a treatment strategy. There remains considerable resistance among family members and clinicians to the complete cessation of all forms of nutritional intake. Accordingly, nutritional support is often continued through subcutaneous or peripheral IV infusion after loss of oral intake, partly in response to family expectations; however, it remains unclear whether IV hydration meaningfully influences survival or the terminal clinical course once swallowing function has irreversibly declined. Accordingly, clarifying the survival prognosis of older adults managed with IV fluids alone after complete loss of swallowing function is essential for informed decision-making and for facilitating goal-concordant discussions with patients and families. In contrast, the prognosis of patients receiving IV fluid therapy alone remains unclear. Although small-scale studies have been reported, there are no data on survival duration in patients who subsequently develop impaired swallowing function, become unable to take oral intake, and receive only IV fluids. This study aimed to describe survival after the initiation of nil per os (NPO) among older adults who lost swallowing function and were managed with IV fluids alone, and to examine differences according to terminal clinical course.

## Materials and methods

Study design

This retrospective observational study was conducted at Tenshindou Hetsugi Hospital between April 2015 and March 2025. The study included patients who were hospitalized due to pneumonia, subsequently recovered, but developed irreversible swallowing impairment, were unable to take food orally, and were managed under an NPO policy until death. Patients who died before recovery from the pneumonia that led to hospitalization were excluded. Because survival prognosis was expected to vary according to the terminal clinical course, patients were classified into three groups based on the principal cause of death: a senility group, a recurrent pneumonia group, and an other-cause group. The senility group was defined as patients managed under an NPO policy who received only IV fluids and died without evidence of acute conditions such as cardiac events or recurrent pneumonia. The clinical course in this group was typically gradual, with decline leading to death without a distinct acute terminal event. Recurrent pneumonia was defined as the development of pneumonia after a period of NPO following treatment, which ultimately resulted in death. The diagnosis of pneumonia was based on clinical symptoms in conjunction with imaging findings. The most frequent cause of death in the other-cause group was sudden cardiac death, followed by septic shock, multiple organ failure, and cerebral infarction, among others.

The primary endpoint was the number of days from the start of NPO to death, and the secondary endpoints were differences in clinicodemographic characteristics and laboratory findings among the three groups. These secondary outcome variables included age, sex, BMI, presence of gastrostomy or NGT feeding, presence of dementia, use of gastric acid suppressants or hypnotics, Activities of Daily Living (ADL), Performance Status (PS), loss of consciousness (Japan Coma Scale), serum albumin, hemoglobin, WBC count, and the presence of comorbidities. Regarding the presence or absence of gastrostomy or NGT feeding, some patients had gastrostomy tubes placed prior to admission, and NGTs were inserted after admission in certain cases; however, neither was used after admission, and no enteral nutrition was administered. In one case, a NGT had already been in place prior to admission, but enteral nutrition was not administered through it. Because ADL has been reported to correlate with PS [11], PS was used as the indicator of functional status in this study. Accordingly, ADL status was defined as a bedridden state for patients with PS ≥3, and impaired consciousness was defined as a JCS score of ≥II. This study was approved by the Institutional Review Board of Tenshindou Hetsugi Hospital (Approval No. 2205; September 22, 2025).

Statistical analyses

The mean and median duration from NPO to death (in days) were determined for each group and compared among groups using the Kruskal-Wallis test. Pairwise comparisons between groups were performed using the Bonferroni correction. For comparisons of baseline characteristics among the three groups, continuous variables were compared using the Kruskal-Wallis test, while categorical variables were compared using the chi-square test or Fisher’s exact test (specifically in cases with small sample sizes). A two-sided p value of < 0.05 was considered statistically significant for all tests.

## Results

**Figure 1:**
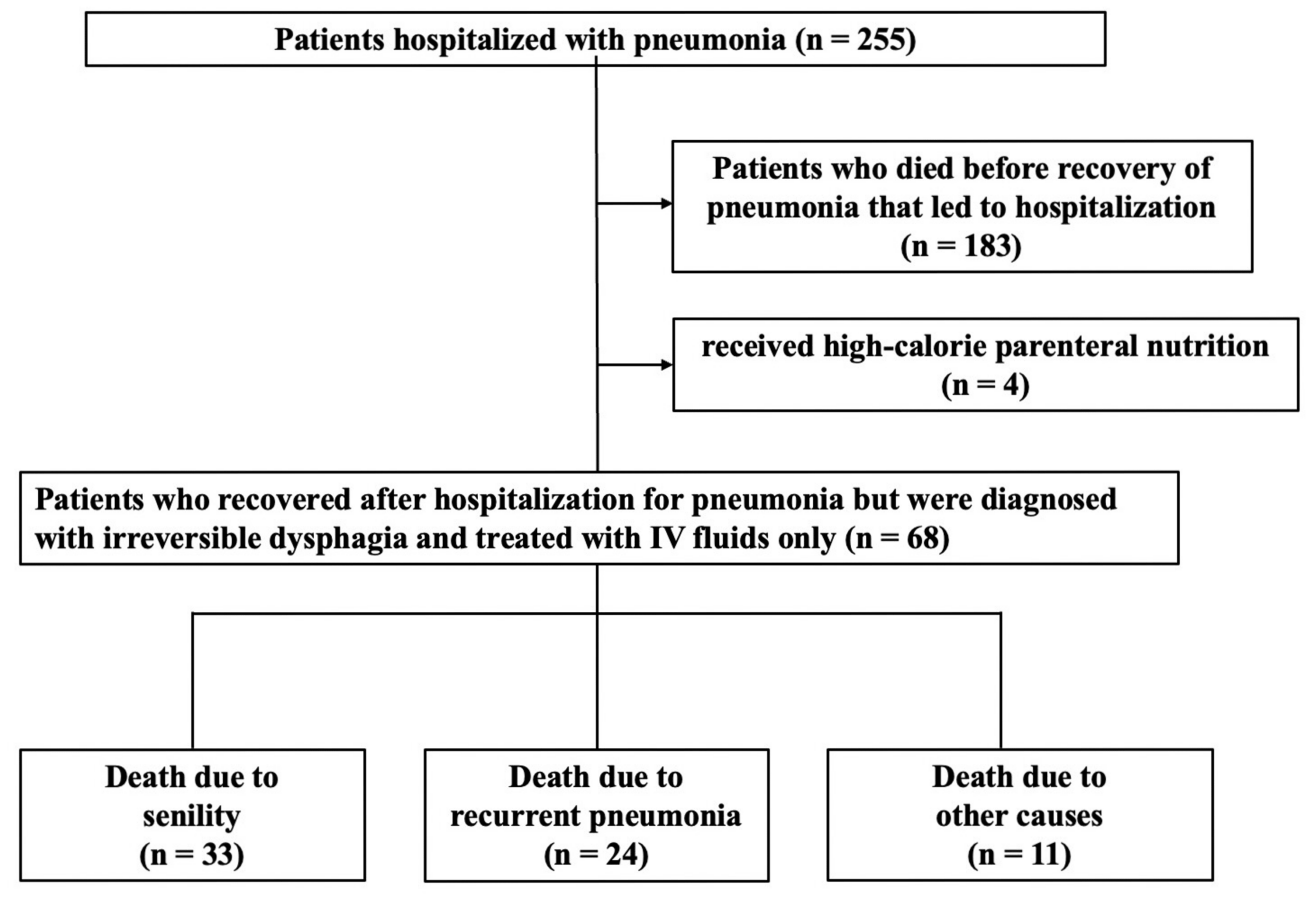
Flow diagram of patient selection and classification. A total of 255 patients were identified during the study period through initial screening, and 68 were included in the analysis after excluding 183 patients who died before recovery from the pneumonia that led to hospitalization and 4 patients who had received high-calorie parenteral nutrition during hospitalization. These patients were classified according to the cause of death into a senility group (n = 33), a recurrent pneumonia group (n = 24), and an other-cause group (n = 11).

**Figure 2:**
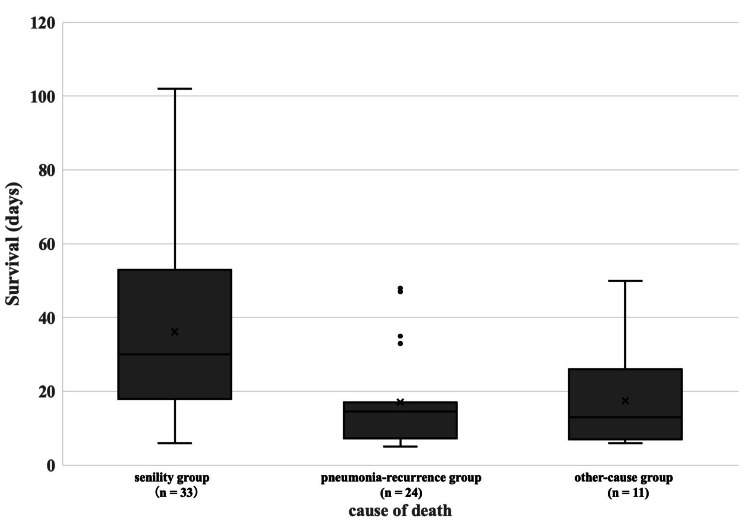
Duration from NPO initiation to death according to cause-of-death group. The box represents the IQR, with the horizontal line indicating the median. Whiskers extend to the most extreme values within 1.5 × IQR, and outliers are shown as individual points. The median survival times were 30.0 days (19.0-47.0) in the senility group, 14.5 days (7.5-17.0) in the recurrent pneumonia group, and 13.0 days (7.0-22.5) in the other-cause group. Comparison of survival duration (days from NPO initiation to death) among the three groups showed a significant difference (Kruskal-Wallis H(2) = 15.41, p < 0.001). In post hoc pairwise comparisons, a significant difference was observed between the senility and recurrent pneumonia groups (p < 0.01), with a longer median survival time in the senility group. NPO: Nil per os.

A total of 255 patients were identified during the 10-year study period through initial screening, of whom 183 were excluded because they died before recovery from the pneumonia that led to hospitalization. The causes of death were multifactorial, making it difficult to provide a detailed breakdown. In addition, four patients who received high-calorie parenteral nutrition via IV hyperalimentation were excluded. The remaining 68 patients were classified according to the cause of death into a senility group (n = 33), a recurrent pneumonia group (n = 24), and an other-cause group (n = 11) (Figure 1). The median duration from NPO onset to death differed significantly among groups (p < 0.01), with significantly longer median survival in the senility group than in the recurrent pneumonia group (30.0 days, 19.0-47.0, vs. 14.5 days, 7.5-17.0; p < 0.01) (Figure 2).

There were no significant differences in baseline clinicodemographic characteristics among groups, except for sex, although BMI and WBC counts were lower in the senility group (Table 1).

**Table 1:** Comparison of clinicodemographic variables among patients stratified by cause of death. Continuous variables were compared using the Kruskal-Wallis test, and categorical variables were compared using the χ² test or Fisher’s exact test, as appropriate. Test statistics are presented as H values for the Kruskal-Wallis test and χ² values for the χ² test. Fisher’s exact test was used for comparisons of gastrostomy or nasogastric tube feeding, chronic respiratory disease, and cerebrovascular disorder.

Variable	Death due to senility group (n = 33)	Death due to recurrent pneumonia group (n = 24)	Death due to other causes group (n = 11)	Test statistic	p-value
Age, years	91 (82-94)	88.5 (82.5-92)	89 (84-98.0)	H = 1.32	0.516
Female	16 (48)	3 (12)	4 (36)	χ² = 9.26	0.008
BMI	15.2 (13.9-17.9)	17.7 (15.0-19.4)	16.2 (15.0-16.9)	H = 3.46	0.177
Presence of gastrostomy or nasogastric tube feeding	5 (15.1)	6 (25.0)	2 (18.1)	Fisher	0.712
Dementia	31 (93.0)	21 (87.5)	9 (81.8)	χ² = 1.50	0.661
Use of gastric acid-suppressing medication	20 (60.6)	16 (66.6)	10 (90.9)	χ² = 3.47	0.184
Bedridden status	33 (100)	23 (96)	11 (100)	H = 3.63	0.162
Loss of consciousness	17 (49)	6 (24)	6 (47)	H = 2.28	0.319
Albumin (g/dL)	2.4 (2.0-2.8)	2.1 (1.9-2.3)	2.2 (1.8-2.6)	H = 2.93	0.231
Hemoglobin (g/dL)	10.3 (8.9-12.1)	10.4 (9.0-12.0)	9.9 (9.3-11.0)	H = 0.48	0.785
WBC (×10³/μL)	8.46 (5.26-10.84)	10.08 (5.78-15.47)	13.70 (8.62-19.39)	H = 5.62	0.06
Chronic respiratory disease	5 (15.1)	8 (33.3)	1 (9.0)	Fisher	0.219
Chronic heart disease	10 (30.3)	6 (25.0)	3 (27.2)	χ² = 0.19	0.934
Cerebrovascular disorder	13 (39.3)	9 (37.5)	1 (9.0)	Fisher	0.177

In addition, chronic respiratory disease was most frequent in the recurrent pneumonia group (Table 1).

## Discussion

Survival durations of older adults with impaired swallowing function managed with IV fluids alone differed significantly according to terminal clinical course. Survival duration after NPO initiation was longer in the senility group than in the recurrent pneumonia group. Longer survival in the senility group may be explained by a more gradual decline without exacerbating events, while recurrent pneumonia may impose an additional physiological burden, leading to more rapid deterioration [12, 13]. However, the present study was descriptive in nature, and the causal mechanisms underlying these differences cannot be determined. Patients in the other-cause group died from heterogeneous clinical conditions such as sequelae of cardiovascular and cerebrovascular events, so interpretation of their shorter survival is more difficult. Nonetheless, these results suggest that preventing recurrent episodes of pneumonia may slow the terminal trajectory of older patients receiving NPO management due to irreversible swallowing dysfunction [14, 15].

While aspiration-related respiratory events may be a major factor influencing survival under NPO management, most other clinical and demographic factors appeared to be of minor importance. In the recurrent pneumonia group, a significantly higher proportion of male patients was observed, suggesting potential sex-related differences in susceptibility to respiratory infections. For instance, a higher prevalence of smoking among men may be one contributing factor [16]. However, this interpretation remains speculative and should be interpreted with caution. BMI tended to be lower in the senility group. Although no causal relationship can be inferred from these observations, we speculate that lower body mass may substantially reduce the energy expenditure required for survival, potentially to the extent that IV hydration alone could be sufficient to meet metabolic demands. Consistent with this notion, one study found that resting energy expenditure was significantly lower among underweight elderly subjects (BMI < 20 kg/m²) [17]. Alternatively, there appears to be a trend toward an association between longer survival and lower WBC counts, which may reflect lower systemic inflammatory status; however, this finding should be interpreted with caution due to the limited sample size.

This study has several methodological limitations. First, this was a single-center retrospective observational study conducted within a specific care environment, and treatment practices such as indications for NPO management, thresholds for withdrawal of enteral nutrition, and the intensity of supportive care may differ across institutions and regions. Therefore, these findings may not be applicable to other geriatric populations receiving NPO management. Second, a large proportion of patients died before resolution of the initial pneumonia. As the analysis was limited to the remaining patients, the quality and intensity of pneumonia management may have introduced selection bias. Third, cause-of-death classification relied on clinical records and physician judgment because autopsies were not available for all cases; consequently, some cases with unrecognized pneumonia or other acute events may have been misclassified as senility-related. Moreover, clinical variables were primarily evaluated at or near the time of admission, and longitudinal changes in nutritional status, inflammatory markers, functional decline, and treatment intensity during the terminal phase were not systematically assessed. Information on potentially important confounders, such as the total volume and composition of IV fluids, use of antimicrobial agents, frequency of aspiration events, and the presence of comfort-focused care decisions, was also limited. In particular, because the volume and composition of IV fluids were not specifically accounted for, variations in fluid administration may have influenced survival trajectories, representing a potentially important confounding factor. Fourth, detailed information regarding dementia severity or stage was not available; therefore, stratification according to cognitive impairment was not possible. Consequently, variability in the degree of cognitive impairment within the senility group may have influenced the observed outcomes, and the extent to which this group accurately represents patients with advanced dementia remains uncertain. Finally, the study population consisted only of patients who died during hospitalization; therefore, the results may not be generalizable to patients discharged alive or to those receiving end-of-life care in community-based settings.

## Conclusions

Among older adults with complete and irreversible loss of swallowing function who received IV fluids alone, without enteral nutrition, survival was significantly shorter in those who developed recurrent pneumonia. These findings may help support prognostic communication and end-of-life care planning, as well as assist clinicians in providing more specific prognostic information to families during informed consent discussions, thereby facilitating shared decision-making regarding end-of-life care, particularly when choosing between peripheral IV hydration and enteral nutrition, including NGT or gastrostomy feeding. In addition, these findings may suggest that prevention of pneumonia might contribute to prolonged survival.
